# A Four-Stage Hybrid Model for Hydrological Time Series Forecasting

**DOI:** 10.1371/journal.pone.0104663

**Published:** 2014-08-11

**Authors:** Chongli Di, Xiaohua Yang, Xiaochao Wang

**Affiliations:** 1 State Key Laboratory of Water Environment Simulation, School of Environment, Beijing Normal University, Beijing, China; 2 State Key Laboratory of Virtual Reality Technology and Systems, Beihang University, Beijing, China; CNRS, France

## Abstract

Hydrological time series forecasting remains a difficult task due to its complicated nonlinear, non-stationary and multi-scale characteristics. To solve this difficulty and improve the prediction accuracy, a novel four-stage hybrid model is proposed for hydrological time series forecasting based on the principle of ‘denoising, decomposition and ensemble’. The proposed model has four stages, i.e., denoising, decomposition, components prediction and ensemble. In the denoising stage, the empirical mode decomposition (EMD) method is utilized to reduce the noises in the hydrological time series. Then, an improved method of EMD, the ensemble empirical mode decomposition (EEMD), is applied to decompose the denoised series into a number of intrinsic mode function (IMF) components and one residual component. Next, the radial basis function neural network (RBFNN) is adopted to predict the trend of all of the components obtained in the decomposition stage. In the final ensemble prediction stage, the forecasting results of all of the IMF and residual components obtained in the third stage are combined to generate the final prediction results, using a linear neural network (LNN) model. For illustration and verification, six hydrological cases with different characteristics are used to test the effectiveness of the proposed model. The proposed hybrid model performs better than conventional single models, the hybrid models without denoising or decomposition and the hybrid models based on other methods, such as the wavelet analysis (WA)-based hybrid models. In addition, the denoising and decomposition strategies decrease the complexity of the series and reduce the difficulties of the forecasting. With its effective denoising and accurate decomposition ability, high prediction precision and wide applicability, the new model is very promising for complex time series forecasting. This new forecast model is an extension of nonlinear prediction models.

## Introduction

Hydrological time series forecasting plays an increasingly important role in the planning, management and optimal allocation of water resources [1]. However, it is still a difficult task due to the complicated stochastic characteristics existing in hydrological series. Further, hydrological processes are affected not only by climate change [2]-[3], including precipitation, evaporation and temperature, but also by human activities and socioeconomic development [4]. Therefore, the hydrological time series always tend to be nonlinear and time-varying [5]. The complex nonlinearity, high irregularity and multi-scale variability make the forecasting of hydrological time series a difficult task. Although many researchers have investigated the problem of hydrological time series forecasting [6]-[7], completely understanding of hydrological processes has not yet been achieved. The forecast accuracy of the current forecasting models is still not high, especially for complex time series.

The current approaches to hydrological forecasting can be divided into two categories: the process-driven models and the data-driven models [8]. Models in the first category mainly consider the internal physical mechanisms of hydrological processes, and they usually need a large amount of data for calibration and validation. However, there is not always enough data available [9]-[10]. The data-driven models are known as black-box methods [11], and they do not consider the physical hydrological process, instead identifying the relationship between the inputs and the outputs mathematically. The data-driven models have been proved to have the advantage of lower demands for quantitative data, better prediction performance and simpler formulation than the process-driven models [12]-[13].

The data-driven models developed in recent decades contain two main categories: traditional statistical techniques and artificial intelligence (AI) tools [14]-[15]. The statistical models can provide good prediction results when the series are linear or near-linear, but they cannot capture the nonlinear patterns hidden in hydrological time series. The nonlinear and AI models include artificial neural networks (ANNs), genetic algorithms (GAs) and support vector machines (SVMs), which provide powerful solutions to nonlinear hydrological forecasting [16]-[18]. However, these AI methods have their own shortcomings and disadvantages. For example, ANNs often suffers from overfitting, and SVMs are usually sensitive to parameter selection.

To overcome the shortcomings of the data-driven models described above and obtain results that are more accurate in forecasting, many hybrid models have been proposed and applied in hydrological series forecasting [19]-[20]. Recently, some hybrid models based on the principle of ‘decomposition and ensemble’ have been proposed. The main purpose of decomposition is to simplify the forecasting process, and the results of ensemble are used to evaluate the forecast performance. Forecast models of this type have already been applied in the field of hydrology research. For example, Kisi [21] used a combination of linear regression model and discrete wavelet transform to predict the river stage. Nourani et al. [22] and Kisi [23] combined the wavelet technique with ANNs to predict rainfall or streamflow time series. Sang [24] developed a method for discrete wavelet decomposition of series and proposed an improved wavelet modeling framework for hydrologic time series forecasting. The results of these studies prove that the ‘decomposition and ensemble’ principle based forecasting methods reduce the difficulty of forecasting and outperform the single models.

However, there are still many problems in prediction when using the ‘decomposition and ensemble’ principle. First, previous researches show that the widely used method presently in decomposing the hydrological time series is the wavelet analysis. However, the effectiveness of the wavelet decomposition is affected by many factors. For example, the accurate wavelet decomposition of series is still a problem and it depends heavily on the choice of wavelet basis function [25]. Second, the results of ensemble are usually defined as the sum of the individual forecasting results [26]. However, this is unreasonable, primarily because the degrees of importance of the intrinsic mode function (IMF) and the residual components are different [27]. In addition, the complicated hydrological time series usually contain noises and show complex characteristics due to the random or uncertain factors of environment [28]-[29], and the hydrological time series forecasting models without considering denoising may influence the prediction accuracy [30]-[31].

To overcome the above three shortcomings, we propose three improvements to promote the prediction accuracy based on the principle of ‘decomposition and ensemble’. The first improvement is to reduce the noises involved in hydrological time series before decomposition, which has a great influence on the forecasting accuracy and may result in over-fitting or underfitting problems [32]. Thus, a novel four-stage hybrid model is developed, consisting of denoising, decomposition, component prediction and ensemble. The second improvement is to adopt more accurate and effective methods for the four stages. During the denoising stage, the empirical mode decomposition (EMD)-based method is employed. The improved EMD method, ensemble empirical mode decomposition (EEMD), is selected as the decomposition tool. Unlike the wavelet analysis and almost other previous decomposition methods, the EMD method describes the local time scale instantaneous frequencies better and does not need any predetermined basis functions [33]-[34]. Another advantage of the EMD-based technique is that it is very suitable for nonlinear and nonstationary time series. The third improvement is utilizing the ANN model instead of simply adding together the individual forecasting results to obtain the ensemble results. Additionally, we choose more reasonable cases with different characteristics and comparison models to thoroughly evaluate the effectiveness of the proposed hybrid-forecasting model. In this paper, we compare our method with other six different methods using six different cases.

The remainder of this paper is organized as follows: Section 2 provides a brief introduction to the methodology of the four stages mentioned above. Section 3 introduces the cases and data used in the evaluation of the proposed model. Section 4 presents and discusses the results of the case study. Finally, Section 5 provides the conclusions of the paper.

## Methods

There are four main stages involved in the proposed hybrid prediction model, i.e., denoising, decomposition, components prediction and ensemble prediction. The methods selected for each stage will be briefly introduced in this section.

### The denoising stage

#### The EMD method

At the beginning of the proposed algorithm, the EMD-based denoising method is employed to reduce the noises contained in the time series. The EMD is an adaptive decomposition method, especially for nonlinear and non-stationary data. The essence of the EMD is to extract IMF components from complex signals. The IMF should satisfy the following two conditions [35]:

- In the whole data set, the number of extrema and the number of zero crossings must either equal or differ at most by one;
- The mean value of the envelope defined by the local maxima and minima should be zero at any point.

For an original time series 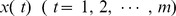, the main steps of the EMD are as follows:

1. Identify all of the local extrema of 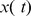.
2. Create the upper envelope 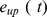
and lower envelope 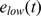 by the cubic spline interpolation, respectively.
3. Compute the mean value 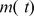
of the upper and lower envelopes: 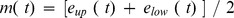.
4. Extract the mean envelope 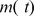 from the signal 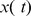, where the difference is defined as 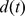:

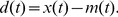
(1)(5) Check the properties of 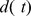:
  - If 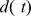 satisfies the requirements (a) and (b), then 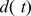 is denoted as the *i* ^th^ IMF, and 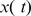 is replaced with the residue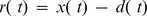. The *i* ^th^ IMF is denoted as 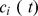, and *i* is the order number of the IMF;
  - If 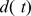 is not an IMF, replace 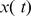 with 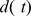.

1. Repeat steps 1–5 until the residue 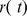 becomes a monotonic function or the number of extrema is less than or equal to one, from which no further IMF can be extracted.

- Finally, the original signal 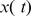 can be expressed as the sum of the IMFs and the residue 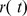:
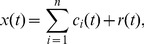
(2)where n is the number of IMFs, 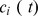 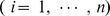 is the ith IMF and 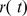 is the final residue. The residue 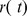 can be seen as the trend of the signal 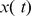 [36]

### The EMD-based denoising method

Many algorithms have been proposed to reduce the noises in series, including spectral analysis, Fourier transforms, wavelet transform and empirical model decomposition (EMD) [37], [38]. Fourier transforms mainly address linear and stationary signals, and the effectiveness of the wavelet-analysis-based denoising method depends on the choice of basic wavelet function and decomposition level [39]. While, the EMD method directly decomposes the original signal into a finite number of components and it performs much better for non-linear and non-stationary signals. In this study, the EMD-based denoising method proposed by Kopsinis and McLaughlin [40] is adopted to reduce the noises of the hydrological time series, and it is briefly introduced as follows:

For the signal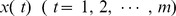, using the EMD method described above, 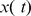
can be expressed as the sum of the IMFs and the residue 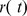:
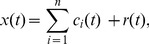
(3)where 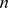 is the number of IMFs and 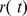 is the residue of 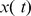. To reduce the noises, a generalized reconstruction of the denoised signal is given as follows:

The core issue of EMD-based denoising is to reconstruct the signal using only the IMFs that contain useful information and discard those that carry primarily noises, i.e., the IMFs that have similar amounts of energy with the noise-only signal. According to the feature of EMD, the power spectra of all of the IMFs excepting the first noise-only IMF exhibit self-similar characteristics and the noise-only IMFs energies linearly decrease in a semilog way. Therefore, the first IMF carries the highest amounts of noise-only energy and noises. In practice, the noise-only IMF energies can be calculated according to [41]:
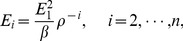
(4)where 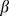 and 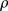 are parameters and depend on the number of sifting iterations used in EMD implementation, which can be estimated in one step with a large number of independent realizations of noise and their corresponding IMFs [41]. In our experiments, the number of sifting iterations is set to 20. The parameters 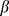 and 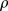 are set to 0.719 and 2.01, respectively. 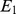 is the energy of the first IMF and can be computed by the variance estimation of the first IMF [42]:



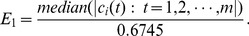
(5)After the analysis and energy calculation of the IMFs, for a noisy signal 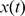, a generalized reconstruction of the denoised signal 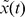 is given as follows:
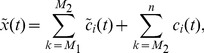
(6)where the parameters 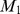 and 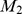 control the number of IMFs used in the reconstruction process [43], which give us flexibility on the exclusion of the noisy low-order IMFs and on the optional thresholding of the high-order ones. It has been empirically found that the good choice of 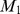 and 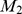 are 2 and 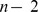, respectively [40]. 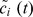
represents the *i* ^th^ thresholded IMF. Inspired by the wavelet thresholding scheme, the thresholded IMF can be obtained by setting the element of each IMF to zero if its amplitude is less than the threshold, and the denoised signal is reconstructed utilizing the high-amplitude elements only [40], [44]. Generally, 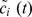 can be obtained by two thresholding schemes: the soft thresholding,



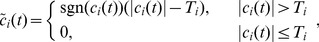
(7)or the hard thresholding,
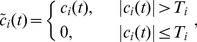
(8)where 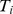 is the threshold of the *i* ^th^ IMF. In our algorithm, we adopt different thresholds 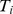 for different IMFs. The adaptive threshold 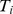 is defined as follows:
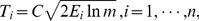
(9)where 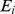 can be calculated using Eq. 4 and Eq. 5. 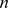 is the number of IMFs. 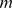 is the length of the original data series. 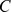 is a constant, which can be selected for each data according to the denoising performance by setting the constant from 0.4 up to 1.4 with step of 0.1 [40]. Actually, according to the results of [40], the optimal constant 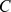 for the denoising algorithm was found to be between 0.6 and 0.8 with a small performance discrepancy for any constant between the above values. Therefore, in our experiments, the constant 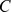 is set to 0.7.

### The decomposition stage

The EMD method has been proven to be an effective decomposition method [35]. However, an obvious drawback of EMD is the frequent appearance of mode mixing. To overcome this defect, Wu and Huang [45] proposed the EEMD method, which is a substantial improvement of EMD and can better separate the scales by adding white noise series to the original time series. Therefore, the EEMD method is selected to decompose the hydrological time series in this stage. The steps of the EEMD are as follows:

1. Add a white noise series to the original data;
2. Decompose the data with added white noise into IMFs using EMD method mentioned above;
3. Repeat step (1) and step (2), but add different white noise series each time;
4. Obtain the ensemble means of corresponding IMFs as the final results.

The added white noise series can cancel each other by taking the average of the corresponding IMFs and the mean IMFs can be close to the original time series by adding noise repeatedly. Therefore, this can significantly reduce the chance of mode mixing and represent a substantial improvement over the original EMD. Nevertheless, how to select the optimal size of the ensemble and the amplitude of the added noise is still an open problem [46]. In fact, the effect of the added white noise can be decreased according to the well-established statistical rule [45]:
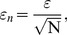
(10)where 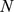 is the number of ensemble members, 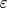
is the amplitude of the added noise, and 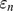
is the final standard deviation of error, which is defined as the difference between the input signal and the corresponding IMF. In this paper, the number of ensemble members is set to 100 and the standard deviation of white noise series is set to 0.2 [47].

### The component prediction stage

ANNs are considered as nonlinear statistical data modeling tools that can simulate the complex relationship between inputs and outputs. In this study, radial basis function neural network (RBFNN), as an ANN technique, is adopted to predict the decomposed IMFs and residual components. The main reason for selecting RBFNN for prediction is that it has a simple structure and a flexible number of neurons [48]. Unlike other types of feed-forward neural networks, the RBFNN has only one hidden layer. Furthermore, RBFNN has a strong approximation ability and fast convergence rate [49].

**Figure 1:**
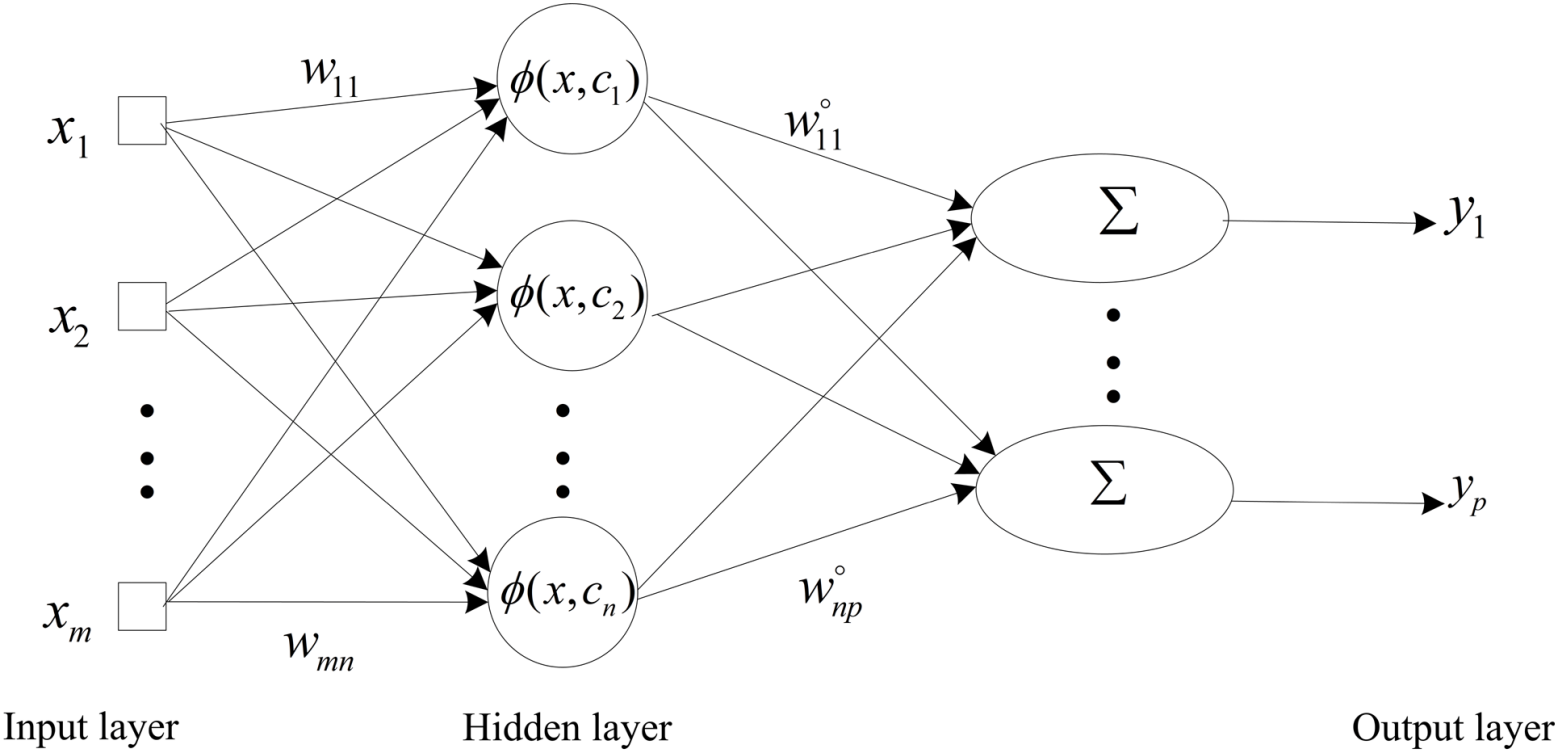
The architecture of the RBFNN. It gives the topological structure of the radial basis function neural network (RBFNN).

RBFNN is a three-layer feed-forward neural network, consisting of an input layer, a hidden layer and an output layer. Fig. 1 shows the architecture of the RBFNN.

In Fig. 1, 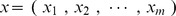 is the input vector; 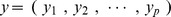 is the output vector; 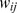
and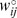 are connection weights. The radial basis function of the hidden layer is denoted as 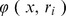 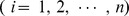. The most popular radial basis function is the Gaussian function:
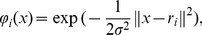
(11)where 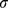
is the standard deviation, 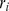
is the *i* ^th^ node center of the hidden layer, and 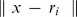 is the Euclidean distance between 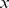 and 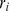.

The main steps of the RBFNN-based forecasting model are shown as follows:

- ***Step 1***. Standardization. The time series 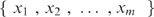 is transformed into 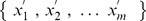 by the following formula:
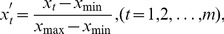
(12)where 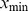
and 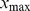
denote the minimum and the maximum of the time series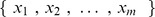.
- ***Step 2***. Forecasting. The forecasting process contains three stages, of which the first is determining the training set and the test set. For the series 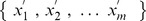, the first
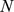
elements are defined as the training set, and the last 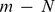 elements are defined as the test set. The second stage is selecting the parameters of the RBFNN. The third stage is determining the error objective function.
- ***Step 3***. Denormalization. Supposing that the forecast result is the series 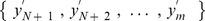, the denormalization formula is as follows:
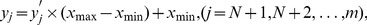
(13)where 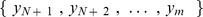 is the final prediction result.

### The ensemble stage

**Figure 2:**
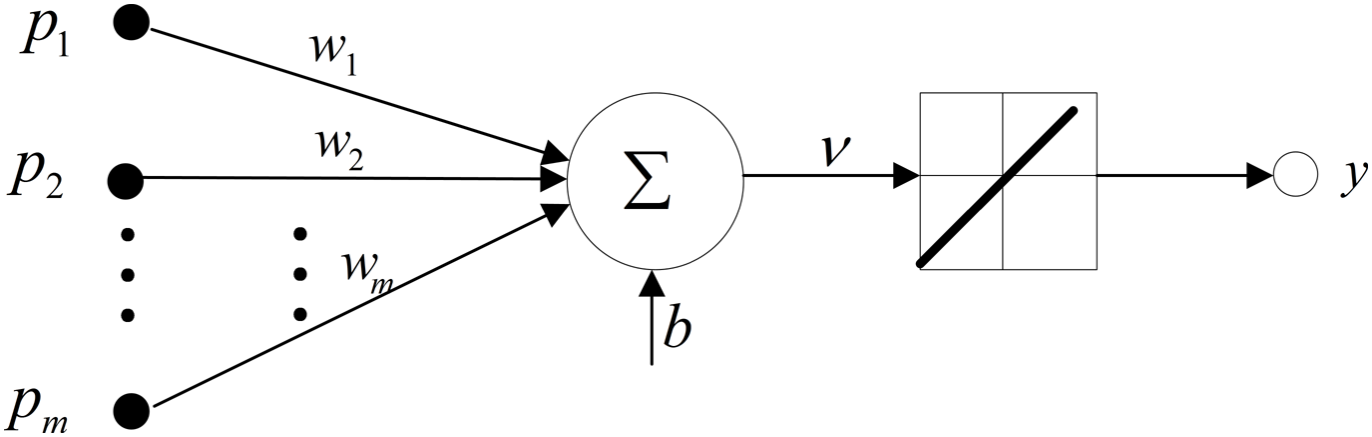
The architecture of the linear neural network.

In the ensemble stage, we adopt a linear neural network (LNN) to integrate the prediction results of the above components, which has a simple structure and the characteristics with fast convergence and high precision. The LNN uses the Widrow-Hoff learning rule known as the least mean square (LMS) to train the neural nets [50]. The architecture of the LNN is shown in Fig. 2.

The LNN algorithm can be expressed as follows:
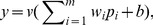
(14)where 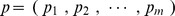 is the input vector, 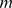 is the number of neuron nodes in the input layer, 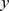
is the target output vector, 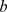
is a bias, 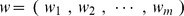 is the connection weight vector, which connects the input variables to the neurons, and 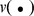 is the transfer function of the single-layer LNN. The mean square error of the LNN neural can be expressed as follows:
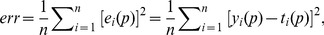
(15)where *err* is the mean square error, 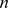
is the number of samples, 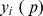 is the network input and 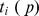 is the target output. The learning rule of LNN is to minimize the mean square error by adjusting the weight vector and the bias, which can be adjusted by the following formulae:
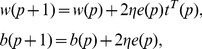
(16)where 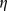 is a learning rate. When 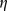 is larger, the learning and convergence speeds are faster, and when 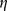 is excessively large, the learning process will be unstable and the error will be bigger. Therefore, to obtain a good convergence to the optimal weight and bias, a suitable learning rate should be chosen.

### The overall process of the proposed four-stage hybrid model

For the original time series 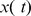
(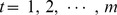), the overall framework of the proposed model is summarized as follows:

1. Denoising. Remove noises in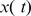 by the EMD interval thresholding-based method, and obtain the denoised time series of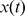, denoted as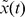.
2. Decomposition. Using the EEMD algorithm, decompose 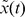
into 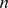 IMFs 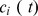 (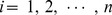) and one residual component 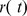.
3. Component prediction. After the decomposition, for each extracted IMFs and residual component, the RBFNN is adopted to model the decomposed components and obtain the corresponding prediction results of each component.
4. Ensemble. The prediction results of all of the extracted IMFs and the residue obtained in the previous step are combined to generate an ensemble result using the LNN model, which can be seen as the final forecasting result of the original time series.

**Figure 3:**
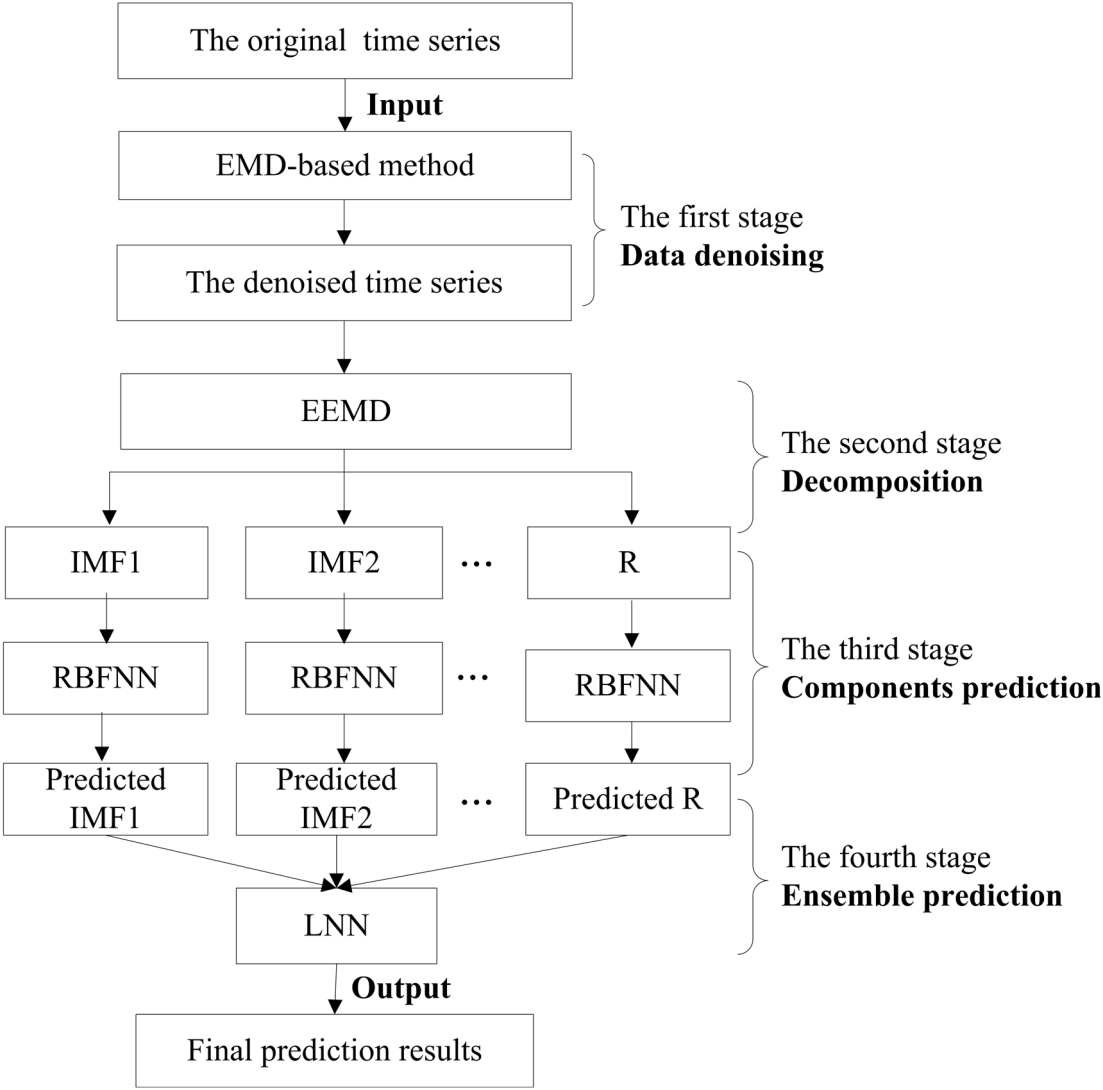
The structure of the proposed EMD-EEMD-RBFNN-LNN model. The proposed model has four stages, i.e., denoising, decomposition, component prediction and ensemble. The methods used in the four stages are empirical mode decomposition (EMD), ensemble empirical mode decomposition (EEMD), radial basis function neural network (RBFNN) and linear neural network (LNN), respectively.

The proposed four-stage hybrid forecasting model has the form of ‘EMD (denoising)-EEMD (decomposition)-RBFNN (component prediction)-LNN (ensemble)’. For convenience, we denote the proposed model as EMD-EEMD-RBFNN-LNN. The structure of the EMD-EEMD-RBFNN-LNN model is shown in Fig. 3.

### Case study and experimental design

To illustrate the effectiveness of our proposed four-stage hybrid forecasting model, several real hydrological cases are studied in this section.

### Study area

The Haihe River Basin (HRB) is selected in this research. As the largest water system in northern China, the HRB is extremely important to China. Influenced by climatic change and human activities, the precipitation and streamfollow have a remarkably interannual and interdecadal variation in the HRB. The precipitation in flood season (June-September) generally accounts for 70%–85% of the annual precipitation. Annual variability of precipitation is also very large. For example, the annual precipitation is more than 800 mm in wet years but only approximately 270 mm in drought years. This leads to complex characteristics in hydrological series. Analyzing the complex characteristics of the hydrological time series and predicting their future trends are significant for water resources planning and ensuring sustainable economic and social development.

### Data description

To thoroughly evaluate the effectiveness of the proposed hybrid forecasting model, six cases with different characteristics are analyzed. The time series of the six cases are denoted by S1, S2, S3, S4, S5 and S6, respectively. Table 1 provides the essential information of the six cases, including the lengths, the time ranges, etc. Specifically, the series S1 presents a 62-year (1951–2012) annual mean precipitation data of the whole HRB. The series S2 and S3 both present 62-year (1951–2012) precipitation data measured at the Beijing weather station, but the first one is annual precipitation data and the other is summer precipitation data, which is defined as the sum of the monthly precipitation in June, July and August. The series S4 presents annual runoff data from 1956 to 2000 measured at the Guantai hydrologic station. The series S5 and S6 are monthly runoff data from January 1956 to December 2000, 540 data points in total. S5 is measured at the Xiangshuibao station in the Yang River of the HRB and S6 is measured at the Miyun Reservoir station in the HRB.

**Table 1:** Six cases studied in this paper.

Case	Series	Station	Type	Length	Time range
Case 1	S1	44 meteorological stations in HRB	Annual mean precipitation of HRB	62	1951–2012
Case 2	S2	Beijing	Annual precipitation	62	1951–2012
Case 3	S3	Beijing	Summer precipitation	62	1951–2012
Case 4	S4	Guantai	Annual runoff	45	1956–2000
Case 5	S5	Xiangshuibao	Monthly runoff	540	Jan.1956 to Dec. 2000
Case 6	S6	Miyun Reservoir	Monthly runoff	540	Jan.1956 to Dec. 2000

**Figure 4:**
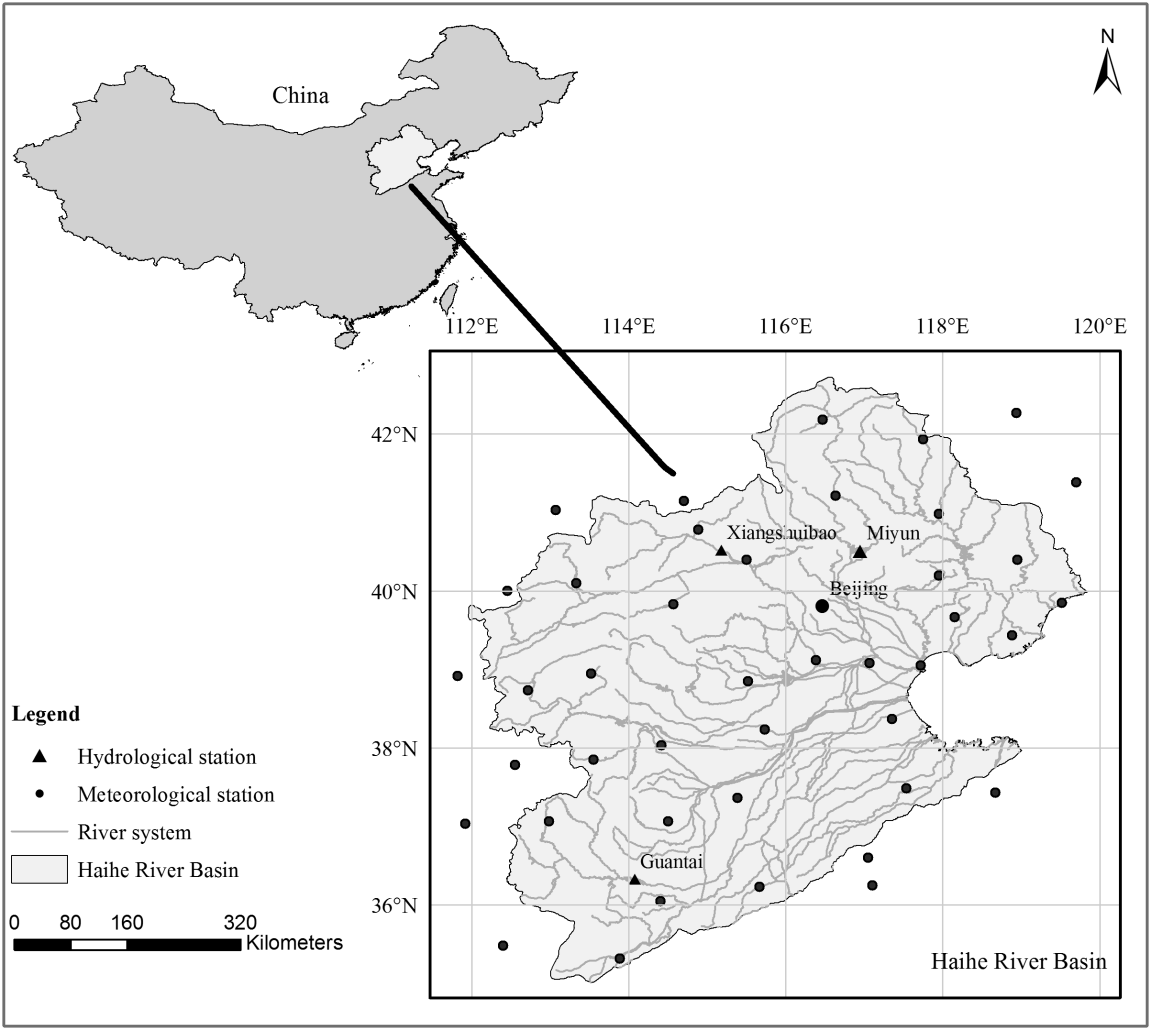
The selected stations of the Haihe River Basin. This figure shows the locations of the 3 hydrological stations (Guantai, Xiangshuibao, Miyun Reservoir) and 44 meteorological stations (including Beijing). The precipitation data of the 44 meteorological stations are used to compute the annual mean precipitation of HRB.

For the six cases, the precipitation data are collected from the China meteorological data sharing service system, and the runoff data are extracted from China’s hydrological yearbook. It should be noted that the data for S2, S3, S4 and S5 are directly collected from the observed stations, as shown in Fig. 4. The annual mean precipitation data of S1 are calculated from the precipitation data of the 44 meteorological stations (shown in Fig. 4) based on the Thiessen polygon theory in Geographic Information System (GIS) software ArcGIS 9.3. The main reason of selecting the six time series is that they have different spatial and temporal scales, including not only data on a whole-basin scale (S1) but also data for single hydrological stations (S2, S3, S4, S5). Additionally, they also have different time scales, including yearly, monthly and seasonal data. Selecting data of different types is helpful for validating the applicability of the model.

### Model evaluation

#### Comparison models

To assess the effectiveness of the proposed EMD-EEMD-RBFNN-LNN hybrid prediction framework, we compared our method with other forecasting approaches. In our experiments, four types, altogether six comparison methods are set. These comparison methods are set in accordance with the following three purposes: (1) To verify the roles of the denoising and decomposition stages in improving the prediction performance, four types of comparison models are set: C1, C2, C3 and C4. (2) To verify the validity of the method selected in each stage of C4, other three hybrid prediction methods with the type of C4 are given. (3) To verify the effectiveness of the ANNs model compared with traditional statistical models, the ARIMA method is selected as a comparison with RBFNN in the prediction stage. Based on the three points above, the comparison models of EMD-EEMD-RBFNN-LNN are set as shown in Table 2. The detailed descriptions of the comparison models in Table 2 are given as follows:

**Table 2:** Four types of comparison models.

Models	Type	Style	Stages	Methods
C1	Single prediction	One-stage	Prediction	RBFNN (ARIMA)
C2	Hybrid model	Two-stage	Denoising-prediction	EMD-RBFNN(ARIMA)
C3	Hybrid model	Three-stage	Decomposition-prediction-ensemble	EEMD-RBFNN(ARIMA)-LNN
C4	Hybrid model	Four-stage	Denosing-decomposition-prediction-ensemble	EMD-EEMD-RBFNN-ADD
				EMD-EEMD-ARIMA-LNN
				EMD-WA-RBFNN-LNN
				**EMD-EEMD-RBFNN-LNN**

The first kind of comparison model C1 is set as a one-stage single prediction model and it predicts the trend of the original time series directly. For comparison, the three types of comparison models C1, C2 and C3 adopt the same approaches with C4 in the corresponding stages. Therefore, the method used by C1 is RBFNN or ARIMA, and the ARIMA method [51] is employed as the comparison method to test the effectiveness of the ANNs method. C2 is set as a two-stage hybrid model. The first stage is to reduce the noises in the original time series and the second stage is to predict the trend of the denoised series. Specifically, we utilize the EMD-based denoising method to reduce the noises of the original time series and then predict the trend of the denoised series with the RBFNN (or ARIMA) method, which is denoted as EMD-RBFNN (ARIMA). C3 is set as a three-stage hybrid prediction model. First, decompose the original time series into various components using EEMD method; then, predict the trends of the components with the RBFNN (ARIMA) model; and finally, integrate the prediction results of all the components based on LNN, which is denoted as EEMD-RBFNN (ARIMA)-LNN.

In addition, C4 is a type of four-stage hybrid model proposed in this paper. First, reduce the noises in the original time series; then decompose the denoised time series and predict the trend of each component; and finally, assemble the prediction results of all of the components. The method we employ for each stage of C4 has been introduced in Section 2, the EMD-EEMD-RBFNN-LNN is developed as our final proposed method. To verify the validity of the selected method in each stage of C4, other three hybrid prediction methods with the form of C4 are given as follows: (1) To test the effectiveness of the ensemble method LNN with the traditional simple addition (ADD) method, the first comparison method denoted by EMD-EEMD-RBFNN-ADD is set. (2) The second method is denoted as EMD-EEMD-ARIMA-LNN, which is set to test the effectiveness of the prediction method RBFNN compared with traditional statistic methods, for example, the ARIMA method. (3) The third method is denoted as EMD-WA-RBFNN-LNN, which is set to test the validity of the EEMD method in the decomposition stage, the wavelet analysis (WA) method is employed as a comparison method with EEMD.

### Evaluation criteria

To evaluate the prediction accuracy, the data for the six forecasted series are divided into two parts (Table 3) for calibration and verification, respectively. The mean relative error (MRE), mean absolute error (MAE) and root mean square error (RMSE) are used for evaluating of different prediction methods, respectively, which are defined as follows:
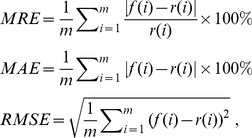
(17)where 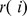 is the real value, 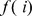 is the forecasted data, and 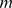 is the size of the data. MRE, MAE and RMSE measure the deviation between the actual and predicted value.

**Table 3:** Data used in the forecasting processes.

Series	Total length	Training data	Validating data
S1	62 years (1951–2012)	52 (1951–2002)	10 (2003–2012)
S2	62 years (1951–2012)	52 (1951–2002)	10 (2003–2012)
S3	62 Summers (1951–2012)	52 (1951–2002)	10 (2003–2012)
S4	45 years (1956–2000)	35 (1956–1990)	10 (1991–2000)
S5	540 months (Jan.1956 to Dec. 2000)	480 (Jan.1956 to Dec.1995)	60 (Jan.1996 to Dec. 2000)
S6	540 months (Jan.1956 to Dec. 2000)	480 (Jan.1956 to Dec.1995)	60 (Jan.1996 to Dec. 2000)

## Results

### Denoising results

**Figure 5:**
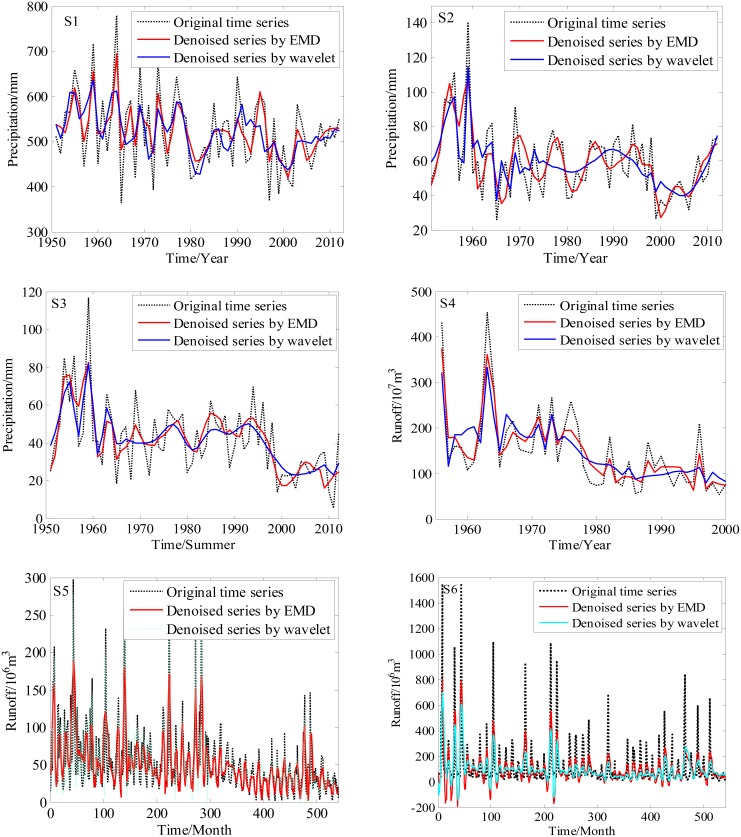
The denoised series of the six hydrological time series. This figure gives the denoising result obtained by the EMD-based method (in red color), as a comparison, the denoising result by the wavelet analysis (in blue color) is also given. It shows much better performances of the EMD-based method in denoising.

The denoising results of the six hydrological time series by the EMD-based method are shown in Fig. 5. The statistical characteristic values, including mean (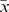), standard deviation (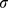), signal-to-noise ratio (SNR) and the root mean square error (RMSE) are used to evaluate the denoising effectiveness, and these are listed in Table 4.
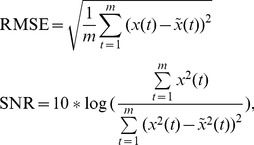
(18)where 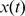
is the original time series, 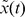
is the denoised time series.

**Table 4:** Statistical characters of the denoising results of the six hydrological time series.

Series	Type	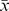	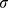	SNR		RMSE	
				EMD	Wavelet	EMD	Wavelet
S1	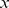	523.6898	86.5809	20.4682	17.6925	50.2834	69.2169
	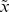 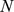	523.6927	50.3798				
		−0.0029	42.9690				
S2	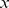	59.2558	20.6472	13.2928	13.21	13.5704	14.23
	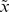	59.4296	16.7573				
	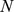	−0.1738	17.2654				
S3	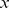	41.8347	19.3510	11.0805	11.0423	12.8528	12.9094
	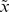	41.8644	14.9367				
	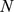	−0.0297	12.9576				
S4	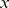	149.5417	88.4751	14.2126	11.0833	33.7328	48.3631
	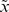	149.2190	68.8477				
	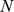	0.3227	34.1124				
S5	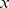	50.4101	38.8410	8.1321	7.721	24.9436	30.6543
	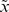	51.2483	33.2675				
	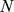	−0.8382	24.9526				
S6	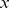	105.94	167.6969	39.03	34.721	122.3210	132.2937
	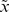	105.48	125.4809				
	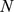	0.47	99.9233				

RMSE (Eq. 18) measures the differences between two series; actually, the smaller the RMSE is, the better the performance that can be obtained by denoising. The SNR (Eq. 18) quantifies how much a signal has been corrupted by noises. Table 4 shows the statistical characteristic values. In Table 4, 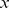, 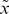 and
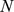
are the original time series, denoised time series and noisy time series, respectively. From Table 4, we can see that the mean values of the denoised and original series are similar. Further, the statistical values of wavelet analysis are also listed in Table 4, and its denoised results are shown in Fig. 5. Compared with the wavelet analysis, the SNR is larger and the RMSE is smaller in the EMD-based denoising method. Therefore, the EMD-based method shows much better performance in denoising, which can be clearly seen from Fig. 5.

### Decomposition results and complexity analysis

**Figure 6:**
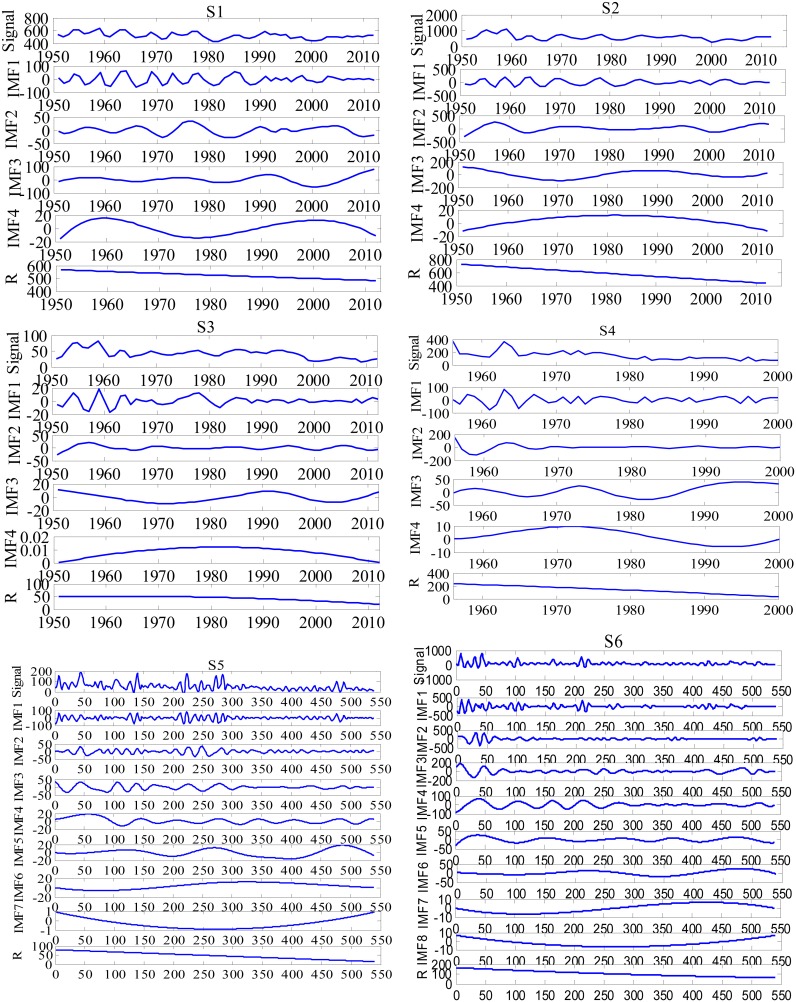
The decomposition results of the six denoised hydrological time series. The six series are decomposed into several IMFs and one residue. The IMFs are listed in the order from the highest frequency to the lowest frequency.

Using the EEMD method, the six denoised hydrological time series obtained above are decomposed into several independent IMFs and one residue. For convenience, the *i* ^th^ IMF is denoted as IMFi (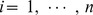), and 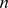 is the number of IMFs. The decomposition results are illustrated in Fig. 6, which lists all of the IMFs in order from the highest frequency to the lowest frequency, and
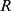
is the residual component that maintains the trend of the original time series. It is easy to see that the series S1, S2, S3 and S4 are decomposed into four independent IMFs and one residue, whereas S5 and S6 are decomposed into seven and eight independent IMFs and one residue, respectively. The decomposition results indicate that the hydrological time series have complex multi-scale characteristics.

**Figure 7:**
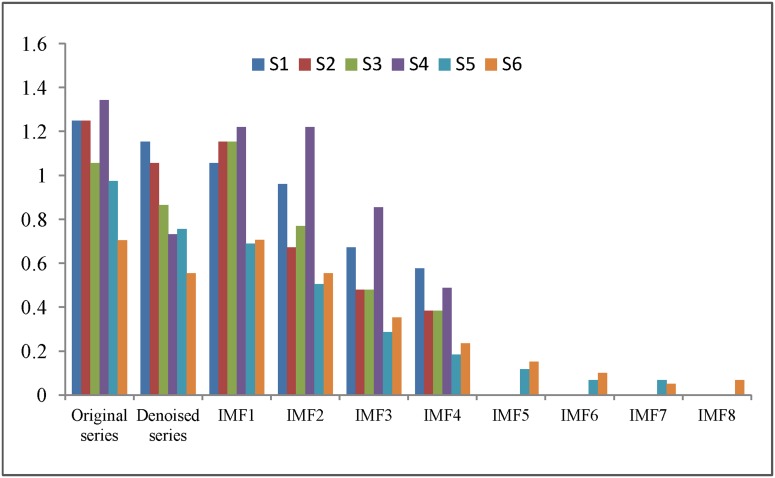
The Lempel-Ziv complexity of the six hydrological time series. It shows the Lempel-Ziv complexity (LZC) of the six original series, denoised series and the IMFs.

In this study, the complexities of the original hydrological time series and the IMFs obtained by decomposition are measured with the Lempel-Ziv complexity (LZC) theory, which has been used in multiple contexts and is considered an effective model for the measurement of complexity and randomness [52]-[53]. Fig. 7 shows the LZC of the six original series, the denoised series and the IMFs. From Fig. 7, we can see that most of the LZC of the original time series in the six cases are larger than 1, which indicates that the hydrological time series have complex characteristics [54]. The LZC values for the denoised time series are smaller than those for the original time series, meaning that the denoising process reduces the complexity of the original sequences. Fig. 7 also shows that the denoised series are decomposed into several IMFs with smaller LZC values, and they decrease from the highest frequency to the lowest frequency; furthermore, most of the LZC values of the IMFs are much smaller than those for the undecomposed series. Therefore, decomposition greatly reduces the difficulty of forecasting.

### Forecasting results

For each extracted IMF and residual component, the RBFNN is adopted to forecast the decomposed components. Similarly, the ARIMA method is employed as a comparative forecast model; that is, each component is predicted by both RBFNN and ARIMA methods. One important part of RBFNN is the determination of the neural nodes of the layers, and the other is the selection of network parameters. In this study, the RBFNN is trained by the toolbox *newrb* in Matlab, which has the format: *net  =  newrb (P, T, goal, spread, MN, DF)*, where the ‘goal’ and ‘spread’ are two important parameters. The neural node of the output layer is set as 1, and the number of neurons of the input layer is determined by training for many cycles; it does not need to select the neural node of the hidden layer because *newrb* adds neurons to the hidden layer adaptively. In an ARIMA (p-d-q) model, the best ARIMA model for each training sample is determined based on the Schwarz Criterion [55].

All of the IMFs and residual components of the six hydrological time series are predicted. Taking S1 and S5 as examples, Tables 5 and 6 show the optimal structure and prediction error of the RBFNN and ARIMA models when modeling the IMFs of the denoised series. The results of the prediction errors in both tables show that the RBFNN performs better than the ARIMA method. Furthermore, with the frequency of each component gradually reduced from IMF1 to the residue *R*, the prediction accuracy of each component gradually increases from IMF1 to *R*, which further implies that simply adding the forecasting results of the IMFs in the ensemble stage is unreasonable and inaccurate.

**Table 5:** The prediction model structure of IMFs of the case 2.

IMFs	RBFNN structure	ARIMA structure	Prediction error						
	n-goal-spread	p-d-q	RBFNN			ARIMA			
			MRE	RMSE	MAE		MRE	RMSE	MAE
IMF1	8–0.01–8	4–1-2	0.7668	2.3416	1.5732		1.0767	2.4528	1.8038
IMF2	5–0-6	2–0-2	0.0714	0.6647	0.5764		0.0587	0.4410	0.3536
IMF3	4–0-5	1–0-6	0.0907	0.2816	0.1683		0.0852	0.2344	0.1975
IMF4	5–0-3	2–0-1	0.00001	0.00002	0.0001		0.0011	0.0001	0.0002
R	4–0-1	2–0-0	0	0	0		0	0	0

**Note**: n-number of the neuron nodes in the input layer.

**Table 6:** The prediction model structure of IMFs of the case 5.

IMFs	RBFNN structure	ARIMA structure	Prediction error						
	n-goal-spread	p-d-q	RBFNN			ARIMA			
			MRE	RMSE	MAE		MRE	RMSE	MAE
IMF1	7-0.005-6	4-1-2	0.8738	2.4513	1.7132		0.9408	2.2649	1.712
IMF2	6-0.01-8	4-0-4	0.1135	2.3094	0.2069		0.0981	0.3189	0.2381
IMF3	8-0.005-4	4-0-1	0.0189	0.2967	0.0162		0.0074	0.0235	0.0156
IMF4	8-0-7	4-0-3	0.0003	0.028	0.0015		0.0002	0.0013	0.0009
IMF5	8-0-4	1-0-4	0.00007	0.002	0.0005		0.03	0.0917	0.0816
IMF6	8-0-7	1-0-8	0.0028	0.0006	0.0008		0.0008	0.0004	0.0004
IMF7	8-0-4	1-0-8	0	0.0008	0			0.0004	0.0004
R	8-0-1	2-0-0	0	0	0		0	0	0

**Note**: n-number of the neuron nodes in the input layer.

**Figure 8:**
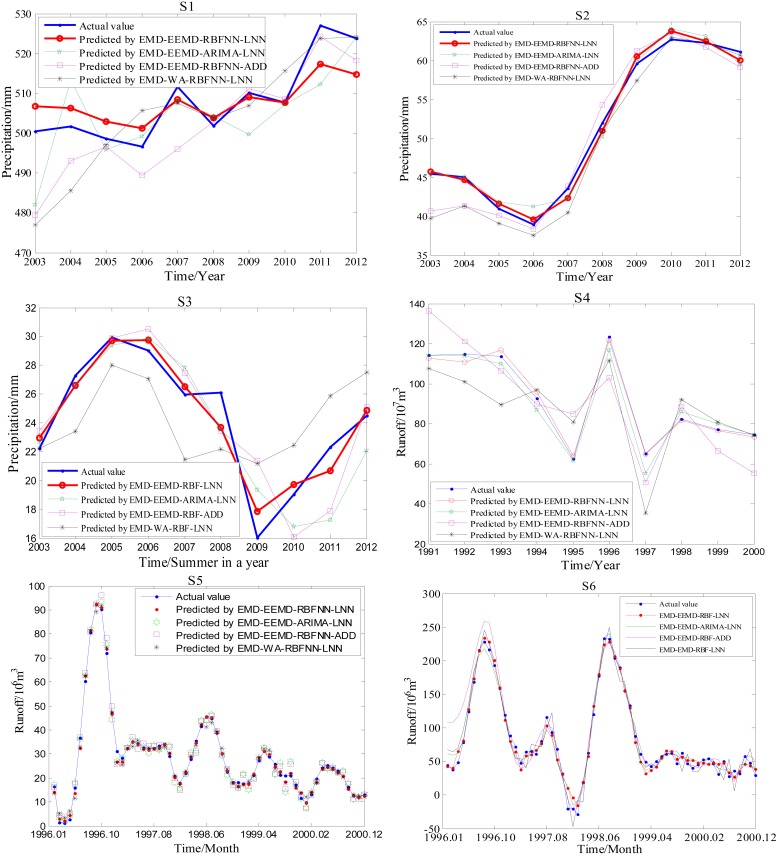
Prediction results of the six series by using the proposed four-stage hybrid forecasting model and its three comparison methods. The proposed four-stage model has the form ‘denoising-decomposition-component prediction-ensemble’. This study utilizes EMD-based denoising method to denoise and decompose the denoised time series by EEMD, then predicts the IMFs by RBFNN and integrates the predicted results by LNN i.e. it has the form ‘EMD-EEMD-RBFNN-LNN’. As a comparison, the prediction results of its three comparison models (in different colors) are also given in this figure.

The results of evaluating the forecasting of six cases are shown in Table 7, where the values in brackets are the prediction results of the ARIMA method as a comparison with the RBFNN model, the text in bold shows the results of the proposed EMD-EEMD-RBFNN-LNN model, and the other values are its comparison models. Fig. 8 shows the prediction results of the six cases by using the four-stage hybrid forecasting model, which contains the EMD-EEMD-RBFNN-LNN method and its three comparison models: EMD-EEMD-RBFNN-ADD, EMD-EEMD-ARIMA-LNN and EMD-WA-RBFNN-LNN.

**Table 7:** Evaluation of the forecasting of the six cases.

Series	Models	Method	Error		
			MRE	RMSE	MAE
S1	C1	RBFNN (ARIMA)	0.0576 (0.0682)	39.4242 (40.6552)	30.6943 (34.9074)
	C2	EMD-RBFNN(ARIMA)	0.0169 (0.0364)	12.1415 (22.6583)	8.5578 (18.3649)
	C3	EEMD-RBFNN(ARIMA)-LNN	0.0521 (0.0519)	35.7531 (36.7028)	25.5369 (27.3512)
	C4	EMD-EEMD-RBFNN-ADD	0.013	9.257	6.6003
		EMD-EEMD-ARIMA-LNN	0.0132	9.1143	6.6901
		EMD-WA-RBFNN-LNN	0.0141	10.0093	7.0872
		**EMD-EEMD-RBFNN-LNN**	**0.0088**	**5.3904**	**4.4765**
S2	C1	RBFNN (ARIMA)	0.1736 (0.2069)	11.6159 (15.3111)	9.3125 (11.5839)
	C2	EMD-RBFNN(ARIMA)	0.0856 (0.086)	5.1704 (5.9117)	4.3946 (5.0769)
	C3	EEMD-RBFNN(ARIMA)-LNN	0.1531 (0.1251)	9.4989 (7.482)	6.9986 (6.4249)
	C4	EMD-EEMD-RBFNN-ADD	0.036	2.2572	1.7702
		EMD-EEMD-ARIMA-LNN	0.022	1.2721	1.0546
		EMD-WA-RBFNN-LNN	0.0321	2.0572	1.6702
		**EMD-EEMD-RBFNN-LNN**	**0.0212**	**1.1345**	**1.0118**
S3	C1	RBFNN (ARIMA)	0.5236(0.5431)	12.1047 (14.2315)	10.7881 (12.3121)
	C2	EMD-RBFNN(ARIMA)	0.154 (0.1631)	4.6591 (5.6123)	3.1207 (4.1734)
	C3	EEMD-RBFNN(ARIMA)-LNN	0.2943 (0.3151)	5.2861 (7.1241)	4.4373 (5.4321)
	C4	EMD-EEMD-RBFNN-ADD	0.1004	2.6518	2.0959
		EMD-EEMD-ARIMA-LNN	0.0908	2.4205	1.9865
		EMD-WA-RBFNN-LNN	0.1379	3.4334	3.1274
		**EMD-EEMD-RBFNN-LNN**	**0.0441**	**1.1946**	**0.9799**
S4	C1	RBFNN (ARIMA)	0.3257 (0.4295)	46.9568 (45.5323)	30.6295 (38.6238)
	C2	EMD-RBFNN(ARIMA)	0.1835 (0.2097)	18.2246 (23.2594)	15.4026(16.717)
	C3	EEMD-RBFNN(ARIMA)-LNN	0.2287 (0.2412)	24.6292 (30.2315)	18.0103 (21.5612)
	C4	EMD-EEMD-RBFNN-ADD	0.1416	14.3944	12.2792
		EMD-EEMD-ARIMA-LNN	0.0406	4.6091	3.4564
		EMD-WA-RBFNN-LNN	0.1449	15.1783	12.217
		**EMD-EEMD-RBFNN-LNN**	**0.0175**	**2.0635**	**1.6963**
S5	C1	RBFNN (ARIMA)	0.3696 (0.3821)	18.5852 (20.5852)	10.8025 (13.8025)
	C2	EMD-RBFNN(ARIMA)	0.1322 (0.1271)	2.4513 (2.8773)	1.8826 (2.1834)
	C3	EEMD-RBFNN(ARIMA)-LNN	0.2781 (0.2823)	11.1481 (12.1182)	8.0307 (9.5313)
	C4	EMD-EEMD-RBFNN-ADD	0.1685	2.4647	1.8824
		EMD-EEMD-ARIMA-LNN	0.1229	2.347	1.8145
		EMD-WA-RBFNN-LNN	0.1633	1.9216	1.4997
		**EMD-EEMD-RBFNN-LNN**	**0.0871**	**1.3346**	**0.9848**
S6	C1	RBFNN (ARIMA)	0.3621(0.3782)	17.6852 (18.8832)	16.7112 (17.8132)
	C2	EMD-RBFNN(ARIMA)	0.2767 (0.3177)	17.4012 (18.6426)	14.3104 (15.2439)
	C3	EEMD-RBFNN(ARIMA)-LNN	0.2512 (0.2617)	16.2011 (17.3281)	13.2131 (15.4312)
	C4	EMD-EEMD-RBFNN-ADD	0.2494	21.8892	14.6176
		EMD-EEMD-ARIMA-LNN	0.1741	10.5115	8.5526
		EMD-WA-RBFNN-LNN	0.2622	15.4365	12.8694
		**EMD-EEMD-RBFNN-LNN**	**0.1264**	**8.8421**	**7.0021**

## Discussion

Based on Table 7 and Fig. 8, we can get the following conclusions:

1. The proposed EMD-EEMD-RBFNN-LNN model performs the best with the lowest values of MRE, RMSE and MAE for all six cases. For example, the predicted results of S1 show that MRE, RMSE and MAE of EMD-EEMD-RBFNN-LNN are reduced by 37.58%∼84.72%, 46.14%∼86.32% and 36.83%∼85.41%, respectively, when compared to the other comparison models listed in Table 7. It has been shown that the forecasting results of any series by C4 are more accurate than those by C1, C2 and C3, indicating that C4 improves the prediction framework. Through comparing several methods in C4, in Fig. 8, we can see that the deviation between the actual value (in blue) and the predicted value is the smallest for the EMD-EEMD-RBFNN-LNN model (in red). This indicates that the proposed EMD-EEMD-RBFNN-LNN hybrid model has the best prediction performance and improves prediction accuracy.
2. Removing the noise of the original time series before forecasting improves the prediction accuracy. This can be seen from Table 7. Results of MRE, RMSE and MAE of the six cases show that C2 performs better than C1; for example, compared to C1, MRE, RMSE and MAE of C2 are reduced by 43.66%, 61.19% and 49.71% for S4, respectively. The only difference between C1 and C2 is that C2 predicts the time series after noise removal.
3. The decomposition strategy does effectively enhance the prediction accuracy. From the prediction results of C1, C2, C3 and C4, it is clear that the prediction accuracy of C3 is less than the prediction accuracy of C1, and C2 performs worse than C4. For example, compared to C1, the MRE, RMSE and MAE of C3 are reduced by 24.75%, 40.02% and 25.66% for S5, respectively. These results indicate that many multi-scale components with different characteristics existed in the hydrological time series. The decomposition process segregates the multi-scale components from the hydrological time series and predicts the components separately, and this can enhance the forecasting performance.
4. Comparing all of the prediction results, the forecasting precisions of the four models C1, C2, C3 and C4 are higher when using the RBFNN model than when using the ARIMA method. This can be easily seen from Table 7. The values in brackets are the prediction results of the ARIMA method, and they are larger than the values of RBFNN for MRE, RMSE and MAE. For example, MRE, RMSE and MAE of EEMD-RBFNN (ARIMA)-LNN are 0.2943(0.3151), 5.2861(7.1241) and 4.4373(5.4321) for the series S3, respectively. This indicates that the nonlinear AI models are more suitable for prediction than traditional statistical models.
5. Compared with the single prediction model, the hybrid prediction model has better forecasting performance. This is because the forecasting results of the four models C1, C2, C3 and C4 have significant differences. Among them, C1 has the worst forecasting accuracy, while C4 has the best forecasting accuracy, especially for the time series with complex multi-time characteristics, such as S5 and S6.
6. For several hybrid prediction models in C4, the prediction performances are different when using different decomposition and ensemble methods. Compared with other decomposition methods, as in Table 7 and Fig. 8, the proposed EMD-EEMD-RBFNN-LNN algorithm can yield much better prediction performance than the EMD-WA-RBFNN-LNN method, demonstrating that EEMD is much more efficient in decomposition than the WA method. For the ensemble strategy, the performances of EMD-EEMD-RBFNN-LNN and EMD-EEMD-ARIMA-LNN are both better than EMD-EEMD-RBFNN-ADD, indicating that LNN is the more powerful ensemble method.
7. Based on Table 7, it can be easily seen that the proposed four-stage model C4 performs better than the other three comparison models for all six cases. Therefore, the proposed ‘denoising-decomposition-prediction-ensemble’ framework has wide applicability for hydrological time series forecasting.

## Conclusions

Considering the intrinsic complexity of hydrological time series, a new method with four stages, EMD-EEMD-RBFNN-LNN, is proposed for predicting the hydrological time series. The results of six cases show that the proposed hybrid prediction model improves the prediction performance significantly and outperforms some other popular forecasting methods. From the results of the six experiment cases, the following conclusions can be drawn:

1. The proposed EMD (denoising)-EEMD (decomposition)-RBFNN (prediction)-LNN (ensemble) model is significantly superior to all other comparison methods in terms of prediction accuracy, including the models ARIMA, EMD-RBFNN and EMD-WA-RBFNN-LNN, etc.
2. The time series denoising and decomposition enhance the forecasting performance, which suggests that the denoising and decomposition strategies are effective approaches for improving prediction accuracy.
3. The results of the complexity analysis show that the denoising and decomposition stages decrease the complexity of the series and reduce the difficulties of the forecasting.
4. As the nonlinear and non-stationary characteristics existed in the hydrological time series, the nonlinear model EMD-EEMD-RBFNN-LNN is more suitable for prediction than traditional statistic models.
5. The proposed four-stage hybrid model has wide applicability in hydrological time series forecasting as it can improve the prediction performance for several hydrological time series with different characteristics.

As the denoising and decomposition stages can decrease the complexity of the series, enhance the forecasting performance and reduce the difficulties of the forecasting, the proposed model can be utilized as a very promising method for complex time series forecasting, especially for hydrological time series with multiple components and high irregularity. In addition, this new forecast model is also capable of solving other nonlinear prediction problems. Certainly, there are still two problems which need to be further studied: (1) The prediction methods need to be further improved because any black-box model has some defects, such as the choice of reasonable parameters and structures; (2) The present study only considers univariate time series analysis, but some factors affecting hydrological time series such as climate change are not taken into consideration. If these factors can be included into the proposed hybrid prediction model, the forecasting performance will be greatly improved.
